# Circulating levels of IL-6 and TGF-β1 in patients with prostate cancer undergoing radiotherapy: associations with acute radiotoxicity and fatigue symptoms

**DOI:** 10.1186/s12885-022-10255-6

**Published:** 2022-11-11

**Authors:** Katarina Kopčalić, Ivana Z. Matić, Irina Besu, Vesna Stanković, Zoran Bukumirić, Tatjana P. Stanojković, Aleksandar Stepanović, Marina Nikitović

**Affiliations:** 1 Institute for Oncology and Radiology of Serbia, Belgrade, Serbia; 2 Institute for Medical Statistics and Informatics, Faculty of Medicine, University of Belgrade, Belgrade, Serbia; 3 Faculty of Medicine, University of Belgrade, Belgrade, Serbia

**Keywords:** Prostate cancer, Radiation therapy, Acute genitourinary radiotoxicity, Acute gastrointestinal radiotoxicity, Late genitourinary radiotoxicity, Late gastrointestinal radiotoxicity, Fatigue, IL-6, TGF-β1

## Abstract

**Background:**

The goal of research was to investigate the possible relations between serum concentrations of IL-6 and TGF-β1, individual and clinical characteristics, and adverse effects of radiotherapy in patients with prostate cancer: acute and late genitourinary and gastrointestinal toxicity, and fatigue.

**Methods:**

Thirty-nine patients with localized or locally advanced prostate cancer who were treated with radiotherapy were enrolled in this study. The acute radiotoxicity grades and fatigue levels were assessed during the radiotherapy and 1 month after the radiotherapy. Estimation of the late radiotoxicity was performed every three months in the first year, every four months in the second year, and then every six months. Serum levels of IL-6 and TGF-β1 were determined before radiotherapy and after the 25^th^ radiotherapy fraction by ELISA.

**Results:**

The significant positive association between diabetes mellitus and changes in acute genitourinary toxicity grades during the radiotherapy was observed in prostate cancer patients. In addition, patients who were smokers had significantly higher maximum fatigue levels in comparison with patients who were non-smokers. The circulating IL-6 levels were significantly higher after the 25^th^ radiotherapy fraction in comparison with levels determined before radiotherapy. The significant positive correlations between pretreatment TGF-β1 levels and maximum genitourinary toxicity grades and between TGF-β1 levels after the 25^th^ fraction and genitourinary toxicity grades after the 25^th^ fraction, were found. The pretreatment IL-6 concentrations and TGF-β1 concentrations after the 25^th^ fraction were positively correlated with maximum genitourinary toxicity grades. The IL-6 levels after the 25^th^ fraction were positively associated with genitourinary toxicity grades after this fraction. The pretreatment IL-6 concentrations were significantly positively correlated with maximum fatigue scores. The significant positive correlation between IL-6 concentrations and fatigue scores after the 25^th^ fraction was determined. The positive correlations between IL-6 and TGF-β1 concentrations measured after the 25^th^ fraction and maximum fatigue scores were observed.

**Conclusions:**

Our results suggest that serum levels of IL-6 and TGF-β1 might influence the severity of acute genitourinary radiotoxicity and fatigue in patients with prostate cancer. Combining clinical parameters and circulating cytokine levels might be useful for the prediction of adverse reactions to radiotherapy.

**Supplementary Information:**

The online version contains supplementary material available at 10.1186/s12885-022-10255-6.

## Background

Prostate cancer is the second most frequent cancer and the fifth leading cause of cancer death among males worldwide, according to the Global Cancer Statistics 2020 report [1]. Different primary treatment options for prostate cancer, such as surgery, radiotherapy, and hormonal therapy, have increased significantly the survival rate. In recent years the maintenance of a patient’s quality of life has become an important factor influencing therapy decisions [2–5]. Approximately two-thirds of patients with prostate cancer will require radiotherapy either as their initial treatment or later after recurrence or disease progression [6]. Healthy, non-transformed tissue surrounding the malignant tumor is also irradiated, thus causing a wide spectrum of side effects. The development of severe side effects is observed in approximately 10% of patients [7]. Patients with prostate cancer treated with radiotherapy may experience symptoms of acute and late toxicity of the lower gastrointestinal (GI) tract (bowel and rectal toxicity) and genitourinary (GU) tract (urethral, bladder, and prostate gland toxicity) [8]. Acute side effects develop in the rapidly proliferating normal surrounding tissue as a consequence of radiation-induced cell death and inflammation and are commonly reversible [9]. The development of late side effects of radiotherapy is caused by tissue fibrosis, tissue shrinkage, and vascular injury [8, 9]. The dosimetric radiotherapy factors (total dose, dose per fraction, and volume of the irradiated tissue), clinical factors, patient’s individual characteristics, such as age, smoking history, body mass index, previous abdominal surgery, then the presence of comorbidities (diabetes, arterial hypertension, cardiovascular disease), use of neoadjuvant hormone therapy and some drugs, may affect the incidence of radiotoxicity in patients with prostate cancer [10–17]. However, the clinical value of patient-related parameters should be evaluated in additional independent studies.

Cancer-related fatigue (CRF) is one of the most common treatment-related side effects of prostate cancer and may have a negative influence on the quality of life of these patients [18–20]. The fatigue symptoms in patients with cancer could be attributed to different biological factors related to immune and inflammatory response, metabolic, neuroendocrine, and neural processes, in addition to individual differences caused by genetic background [21].

Cytokines, the mediators of an immune and inflammatory response, are released and upregulated in response to ionizing radiation [22, 23]. The radiation-induced inflammatory response and specific changes in the levels of circulating cytokines have been associated with the occurrence of side effects in cancer patients undergoing radiotherapy, such as normal tissue radiotoxicity and fatigue symptoms [24–26]. In our previous studies, we have shown associations between individual, clinical factors, biological parameters, and the risk for development of acute radiotoxicity in patients with prostate cancer treated with radiotherapy [12, 27]. The increased serum levels of IL-6 during the course of radiotherapy in patients with prostate cancer had been reported to be significantly associated with higher grade of acute GU toxicity across radiotherapy [27]. The serum levels of TGF-β1 in patients with prostate cancer tended to increase during radiotherapy [27]. The gene expression levels of TGF-β1 in peripheral blood mononuclear cells were significantly decreased after the last fraction of radiotherapy when compared with those levels before radiotherapy [27]. These results encouraged us to continue the investigation of multiple factors underlying the development of side effects of radiotherapy for prostate cancer.

The aim of the present study was to examine the possible associations between individual and clinical parameters, as well as circulating levels of IL-6 and TGF-β1, and the occurrence of main adverse effects of radiotherapy in patients with prostate cancer – acute and late GU and GI radiotoxicity, as well as fatigue intensity.

## Patients and methods

### Patients

The target population in our study included 39 patients with a histologically confirmed localized or locally advanced prostate cancer who were treated at the Institute for Oncology and Radiology of Serbia from October 2017 to February 2020. 3D conformal radiotherapy (3DCRT) was performed in 27 patients. Since we provided the equipment and started to implement Volumetric Modulated Arc Therapy (VMAT) in our institution, the other 12 patients were treated with this technique. Definitive radiotherapy was performed in 26 patients, while 13 patients received postoperative radiotherapy. Exclusion criteria were: neoadjuvant or concomitant hormonal therapy, the presence of enlarged lymph nodes (N1 stage) detected by imaging methods, the presence of distant metastasis (M1 stage) detected by imaging techniques and previous pelvic irradiation.

The study protocol was approved by the Ethics Committee of the Institute for Oncology and Radiology of Serbia (approval No3348/1–01). The study was carried out according to the principles of the Declaration of Helsinki. The details of the study were explained to the patients and informed consent forms were obtained from the participants.

We obtained individual, clinical, and treatment characteristics for all participants as well as the toxicity scores and fatigue level data. Investigated individual characteristics were as follows: age, smoking status, and alcohol consumption. Clinical characteristics included: medical comorbidities such as diabetes mellitus, hypertension, previous abdominal or pelvic surgery, and other malignancies. Treatment characteristics were related to the type of radiotherapy (definitive or postoperative) and dose-volume groups.

Acute GU and GI radiotoxicities were evaluated weekly according to Acute Radiation Morbidity Scoring Criteria (RTOG/EORTC) modified by Peeters [28], as well in our previous studies [12, 27]. According to Peeters and coworkers, acute side effects occur within 120 days from the start of radiotherapy, whereas side effects occurring from 120 days after the start of treatment were considered late radiation toxicity. Grade of acute radiation toxicity was recorded by the radiation oncologist after every 5 radiotherapy fractions, at the end, and one month after the end of radiotherapy.

As patients didn’t fill the pre-radiotherapy toxicity questionnaires, the symptoms described after 5 fractions could be considered as baseline symptoms, because the dose received up to that point couldn’t cause significant acute toxicity.

Data for late toxicity were collected by patient’s interviews during the subsequent follow-up examinations using Late Radiation Morbidity Scoring Criteria (RTOG/EORTC) modified by Peeters. In the first and second years after radiotherapy, examinations were performed every three and four months, respectively. Thereafter, patients filled out questionnaires six-monthly. The maximum follow-up was 30 months, but further monitoring is ongoing. After five years of radiotherapy completion examinations will be conducted annually. The maximum acute and late GU and GI toxicity grades were recorded for each patient. In addition to collecting data related to toxicity, at each follow-up visit physical examination, PSA determination, and other examinations if necessary (imaging, endoscopy) were performed.

The Expanded Prostate Cancer Index Composite (EPIC) was used to measure the quality of life in prostate cancer patients regarding genitourinary and gastrointestinal symptoms [29]. These questionnaires were filled by eligible participants before radiotherapy, after the 25^th^ fraction, at the end, and 1 month after the end of radiotherapy.

The Brief Fatigue Inventory (BFI) was used for the rapid assessment of fatigue severity using 0–10 scales [30]. The fatigue levels assessed weekly by BFI were divided into categories of “mild” (score 1–4), “moderate” (score 5–6), and “severe” (score 7–10).

### Treatment regimen

Radiotherapy was performed by 3DCRT and VMAT technique, according to the protocol of the Institute for Oncology and Radiology of Serbia, partly described in our previously published articles [12, 27, 31]. Patients treated with definitive radiotherapy received irradiation to different volumes according to the estimated risk of seminal vesicles (SV) and lymph node involvement according to the Roach formula [32]. Those patients were divided in three dose volume groups. The first group was the prostate-only group (P) if the risk for SV involvement was < 15%, the second group was the prostate and seminal vesicle group (P + SV), if the risk SV involvement was ≥ 15% and the risk for lymph node involvement was < 15%. The third group was whole pelvic radiotherapy (WPRT) group if the risk for lymph node involvement was ≥ 15%. Clinical target volume (CTV) and Planning target volume (PTV) were used as standardized nomenclature according to International Commission on Radiation Units and Measurements recommendations ICRU 50, ICRU 62, and ICRU 83 [33–35]. CTV depending of the dose volume group included the whole prostate or included whole prostate with entire seminal vesicle or included whole prostate with entire seminal vesicle and lymph nodes (lnn). CTV Ln encompassed the pelvic lymph node below of bifurcation of *a. iliaca communis* with margin 7 mm around blood vessels. Treatment margins around CTV were defined according our own institutional protocol [12, 27, 31].

The prescribed dose to the ICRU reference volume to cover PTV, in the P only group, was 72 Gy. In the second group the prescribed dose to cover PTV1 (prostate + SV + margins) was 66 Gy and to cover PTV2 (prostate + margins) was 6 Gy. In third group prescribed dose were 44 Gy for PTV1 (prostate + SV + CTVlnn + margins), 22 Gy for PTV2 (prostate + SV + margins) and 6 Gy for PTV3 (prostate + margins). All patients treated with definitive radiotherapy received 72 Gy in 36 fractions regardless it was used 3DCRT or VMAT technique.

Patients who were irradiated with postoperative or salvage radiotherapy had one or two dose volume groups. If pN status was N0 they received 66 Gy to the PTV (prostate bed ± SV). If pN status was Nx or N1 they received 44 Gy to PTV1 (cover pelvic lymph node and the prostate bed ± SV bed) followed by 22 Gy to PTV2 (cover the prostate bed ± SV bed). The SV bed was irradiated in patients with stage pT3b in the pathological report. All patients treated with postoperative or salvage radiotherapy with 3DCRT or VMAT received 66 Gy in 33 fractions [27].

All patients were irradiated with a conventional fractionation regime: 2 Gy daily, 5 days a week. Dose constrains were according to Quantec recommendations [36].

### Determination of serum IL-6 and TGF-β1 concentrations

The concentrations of IL-6 and TGF-β1 in the sera of patients were determined by commercial ELISA kits, according to the assay procedure defined by the manufacturer (Quantikine® ELISA Human IL-6 and Human TGF-β1 Immunoassays, R&D Systems, catalog numbers: D6050, DB100B, respectively). The serum concentrations of cytokines were measured before the start of radiotherapy and after the 25^th^ radiotherapy fraction. The serum samples of patients and dilutions of IL-6 or TGF-β1 standards were added in duplicate to 96-well ELISA microplates, precoated with monoclonal antibodies specific for human IL-6 or TGF-β1. After two hours of incubation at room temperature, the plates were washed, IL-6 or TGF-β1 conjugates (polyclonal antibodies specific for antigen conjugated to horseradish peroxidase) were added to all wells and plates were incubated for a further 2 h at room temperature. After the washing steps, the substrate solution containing tetramethylbenzidine and hydrogen peroxide was added to all wells, and the plates were incubated for 20 min or 30 min at room temperature in the dark. The reaction was terminated by adding a stop solution. The optical density was measured at a wavelength of 450 nm using a Multiskan EX Thermo Labsystems microplate reader. The concentrations of IL-6 and TGF-β1 were read from the standard curves generated using four-parameter logistic (4-PL) curve fit.

### Statistical analysis

All data are presented as n (%), mean ± standard deviation or median (range). Statistical hypotheses were tested using: Mann–Whitney U test and Wilcoxon test. Spearman rank-order correlation coefficient was used for examinations of the possible correlations between the tested variables. Examination of the relations of the dependent variable in repeated measurements with possible predictors, was done using linear mixed effects modeling approach using *nlme* package for the R statistical computing environment (R Core Team, Vienna, Austria) [27].

The level of statistical significance was set at 0.05.

## Results

### Patient characteristics, radiation-induced toxicity and fatigue

The characteristics of the study patient cohort are shown in Table 1.

**Table 1:** Patient, clinical, tumor, and treatment characteristics

**Number of patients (n)**	39
**Age; mean ± sd**	70.7 ± 6.7
**Smoking status; n (%)**	29 (74.4%)
**Alcohol consumption; n (%)**	
Yes, regular	1 (2.6%)
Periodically	23 (59.0%)
No	15 (38.5%)
**Medical comorbidities; n (%)**	
*Diabetes mellitus*	7 (17.9%)
Hypertension	28 (71.8%)
Operations/abdominal	17 (43.6%)
Other malignancies	36 (92.3%)
**PSA before diagnosis; median (min – max) ng/mL**	10.0 (3.9–38.8)
**PSA before radiotherapy; median (min–max) ng/mL**	0.6 (0.1–14.3)
**T stage; n (%)**	
T2	27 (69.2%)
T3	12 (30.8%)
**Gleason score; n (%)**	
6	7 (17.9%)
7	30 (76.9%)
8	1 (2.6%)
9	1 (2.6%)
**N stage; n (%)**	
N0	38 (97.4%)
N1	1 (2.6%)
**Type of radiotherapy; n (%)**	
Definitive	26 (66.7%)
Postoperative	13 (33.3%)
**Dose volume groups; n (%)**	
Prostate	5 (12.8%)
Prostate and seminal vesicles	13 (33.3%)
Prostate, seminal vesicles, and lymph nodes	21 (53.8%)
**Risk groups**	
Low risk	5 (12.8%)
Intermediate risk	26 (66.7%)
High risk	8 (20.5%)

Definitive radiotherapy was performed in 26 patients (66.7%). Postoperative radiotherapy was used in 13 patients (33.3%); in 5/13 patients adjuvant radiotherapy was performed, while 8/13 patients were treated with salvage radiotherapy. 3DCRT and VMAT were performed in 27 and 12 patients, respectively. The mean age of the patient group was 70.7 ± 6.7.

**Fig. 1:**
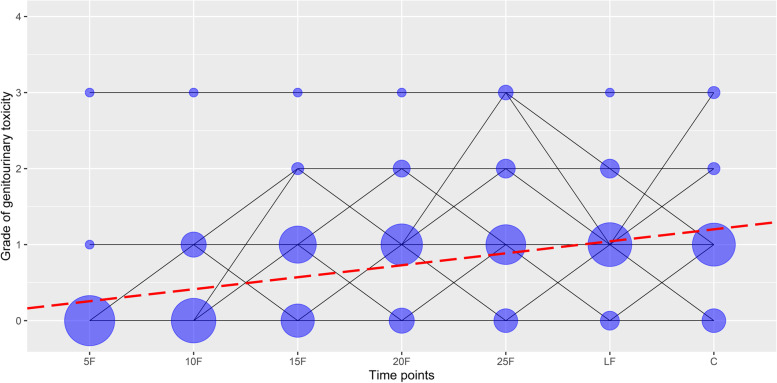
Changes in acute genitourinary toxicity grades at specific time points in patients with prostate cancer treated with radiotherapy: 5F-after the 5^th^ fraction of radiotherapy, 10F-after the 10^th^ fraction of radiotherapy, 15F- after the 15^th^ fraction of radiotherapy, 20F-after the 20^th^ fraction of radiotherapy, 25F-after the 25^th^ fraction of radiotherapy, LF-at the last fraction of radiotherapy, and C-at the first control examination one month after the end of radiotherapy. Lines represent acute genitourinary toxicity grade changes across time. The area of a circle is proportional to the number of patients. At each time point, the sum of the circle area is 100%

The univariate analysis of changes in acute GU toxicity grades at specific time points during the radiotherapy and 1 month after the end of radiotherapy, controlled for type of radiotherapy, demonstrated a statistically significant increase in acute GU toxicity grades over time in patients with prostate cancer (b = 0.158, *p* < 0.001), as presented in Fig. 1. Associations between changes in acute GU toxicity grades across radiotherapy and patient’s individual and clinical characteristics are shown in Table 2. A significant positive association between grades of acute GU toxicity during the course of radiotherapy and the presence of diabetes mellitus in patients with prostate cancer was found (b = 0.540; *p* = 0.007).

**Table 2:** Associations between changes in acute genitourinary toxicity grades across radiotherapy and patient’s individual and clinical characteristics

Parameter	Univariate analysis ^a^	
	**b**	***p***
Smoking status—smokers	0.126	0.494
Alcohol consumption	-0.259	0.116
Diabetes mellitus	0.540	**0.007**
Chronic hypertension	0.107	0.549
Abdominal operation	-0.181	0.255

^a^ multilevel ordinal regression models with the degree of toxicity as the dependent variable, controlled for type of radiotherapy

**Fig. 2:**
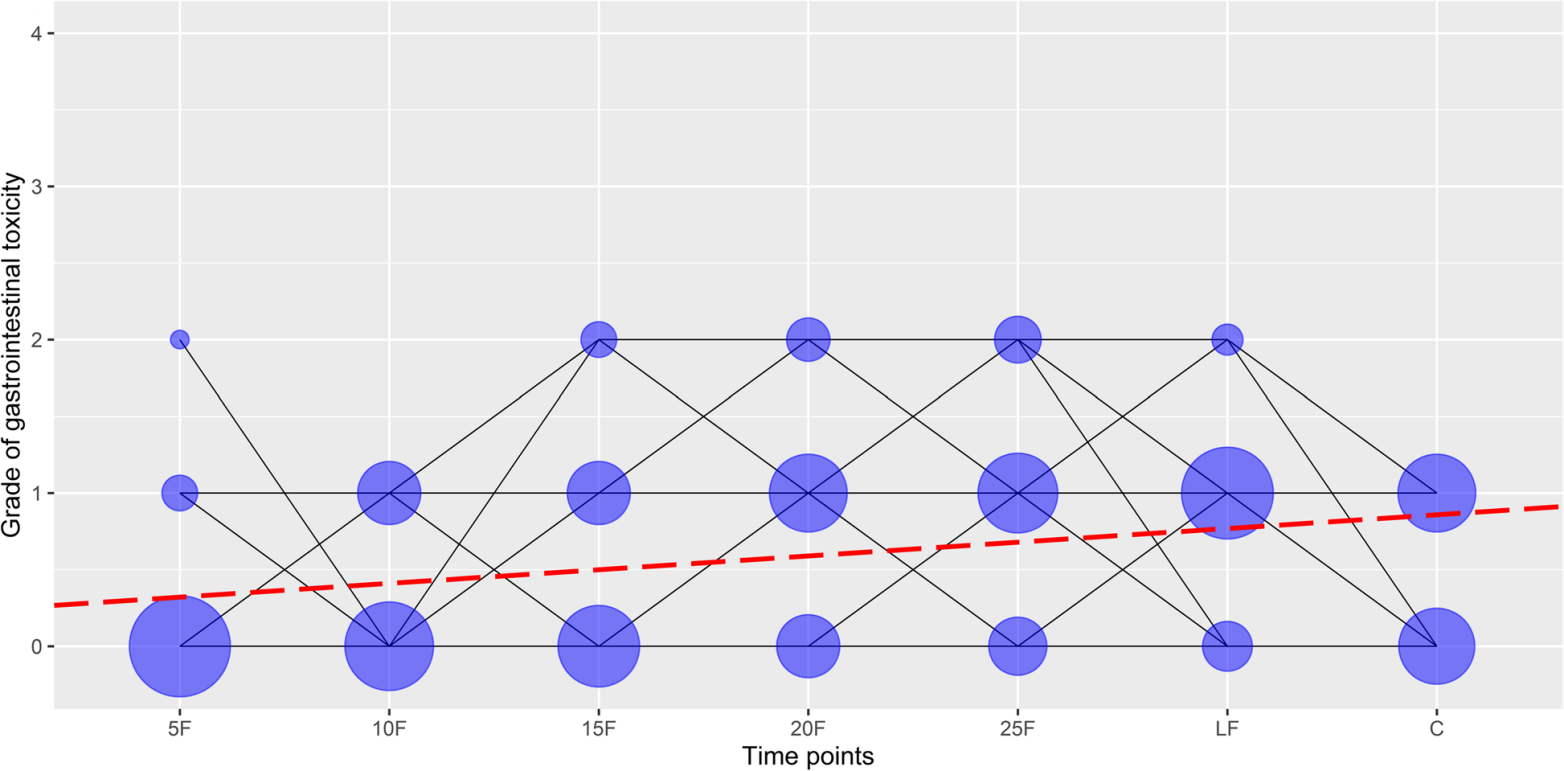
Changes in acute gastrointestinal toxicity grades at specific time points in patients with prostate cancer treated with radiotherapy: 5F-after the 5^th^ fraction of radiotherapy, 10F-after the 10^th^ fraction of radiotherapy, 15F- after the 15^th^ fraction of radiotherapy, 20F-after the 20^th^ fraction of radiotherapy, 25F-after the 25^th^ fraction of radiotherapy, LF-at the last fraction of radiotherapy, and C-at the first control examination one month after the end of radiotherapy. Lines represent acute gastrointestinal toxicity grade changes across time. The area of a circle is proportional to the number of patients. At each time point, the sum of the circle area is 100%

The univariate analysis of changes in acute GI toxicity grades at specific time points during the radiotherapy and 1 month after the end of radiotherapy, controlled for type of radiotherapy, showed a significant increase in acute GI toxicity grades over time in patients with prostate cancer (b = 0.090, *p* < 0.001), as presented in Fig. 2. Results of the examination of possible associations between changes in acute GI toxicity grades across radiotherapy and patient’s individual and clinical characteristics are shown in Table 3. The univariate analysis didn’t show significant associations between changes in acute GI toxicity grades across radiotherapy and individual and clinical characteristics of a patient cohort.

**Table 3:** Associations between changes in acute gastrointestinal toxicity grades across radiotherapy and patient’s individual and clinical characteristics

Parameter	Univariate analysis ^a^	
	**b**	***p***
Smoking status—smokers	0.047	0.729
Alcohol consumption	-0.096	0.432
Diabetes mellitus	0.000	1.000
Chronic hypertension	-0.050	0.704
Abdominal operation	0.102	0.385

^a^ multilevel ordinal regression models with the degree of toxicity as the dependent variable, controlled for type of radiotherapy

**Fig. 3:**
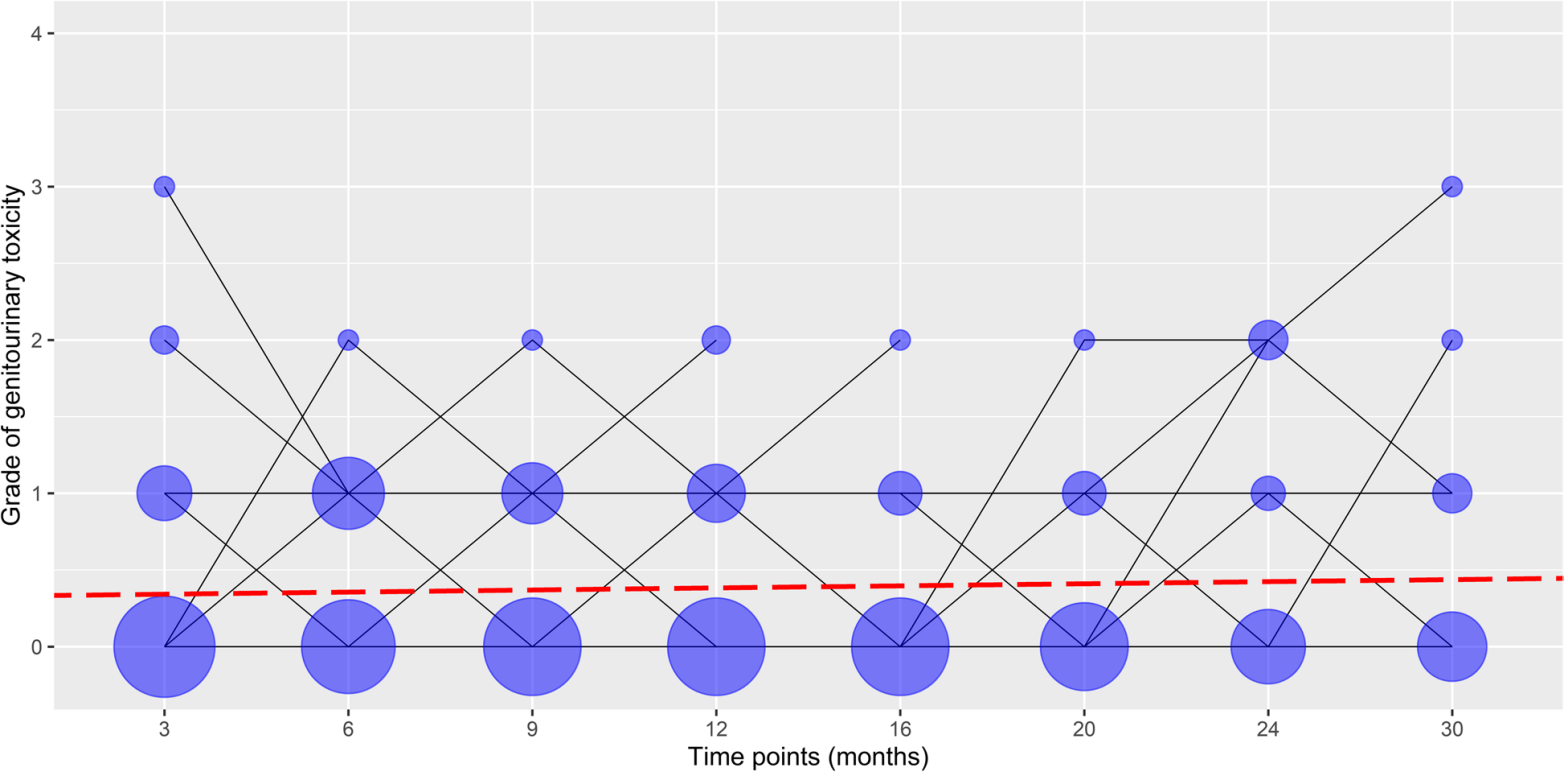
Changes in late genitourinary toxicity grades 3, 6, 9, 12, 16, 20, 24, and 30 months after radiotherapy in patients with prostate cancer. Lines represent late genitourinary toxicity grade changes across time. The area of a circle is proportional to the number of patients. At each time point, the sum of the circle area is 100%

**Fig. 4:**
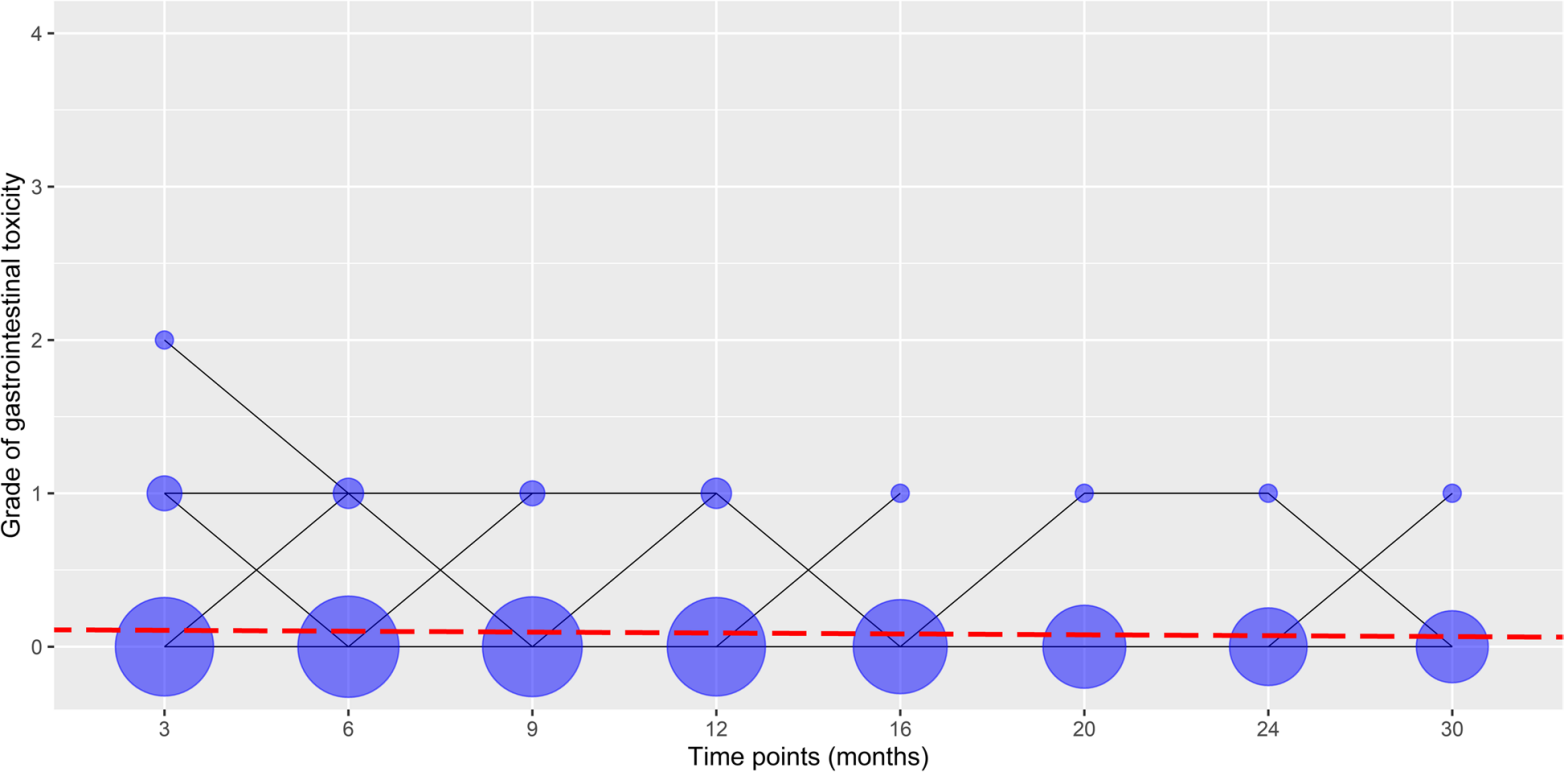
Changes in late gastrointestinal toxicity grades 3, 6, 9, 12, 16, 20, 24, and 30 months after radiotherapy in patients with prostate cancer. Lines represent late gastrointestinal toxicity grade changes across time. The area of a circle is proportional to the number of patients. At each time point, the sum of the circle area is 100%

The univariate analysis of changes in late GU toxicity grades 3, 6, 9, 12, 16, 20, 24, and 30 months after radiotherapy in the patient cohort, controlled for type of radiotherapy, did not show statistically significant changes in late GU toxicity grades over the time course (b = 0.014; *p* = 0.368), (Fig. 3). Within 30 months after radiotherapy, no significant changes in late GI toxicity grades were observed in patients with prostate cancer (b = -0.006; *p* = 0.430) (Fig. 4).

Investigation of a possible influence of patient’s individual and clinical characteristics on maximum fatigue scores determined in patients is shown in Table 4. This statistical analysis showed that prostate cancer patients who were active smokers had a higher maximum fatigue scores during the course of radiotherapy when compared with these scores measured in patients who were non-smokers (*p* = 0.039). The other examined individual and clinical characteristics of patients did not show a significant effect on detected maximum fatigue scores.

**Table 4:** Influence of patient’s individual and clinical characteristics on maximum fatigue scores

Variable	*P* value
Smoking status—smokers	**0.039**
Alcohol consumption	0.977
Diabetes mellitus	0.641
Chronic hypertension	0.656
Abdominal operation	0.729

### Changes in serum cytokine levels

In patients with prostate cancer treated with radiotherapy serum levels of IL-6 and TGF-β1 were measured before the start of radiotherapy and after the 25^th^ radiotherapy fraction. IL-6 serum levels were significantly increased in patients with prostate cancer after the 25^th^ radiotherapy fraction in comparison with those levels determined before radiotherapy (*p* < 0.001) (Table 5). There was no significant difference between serum concentrations of TGF-β1 in patients with prostate cancer before radiotherapy and after the 25^th^ radiotherapy fraction.

**Table 5:** Serum concentrations of IL-6 and TGF-β1 in patients with prostate cancer before radiotherapy and after the 25^th^ radiotherapy fraction

Cytokine	Before radiotherapy	After the 25^th^ radiotherapy fraction	*p*
	**Concentration [pg/mL] median (minimum–maximum)**		
**IL-6**	4.6 (0.8–41.7)	6.3 (2.3–49.3)	** < 0.001**
**TGF-β1**	9152.8 (1600.3–37,421.8)	8104.4 (2465.1–20,718.9)	0.136

### Associations between genitourinary toxicity grade, gastrointestinal toxicity grade, fatigue score, and IL-6 and TGF-β1 concentrations

Examinations of possible correlations between maximum acute GU toxicity grade, maximum acute GI toxicity grade, maximum fatigue score, and serum concentrations of IL-6 and TGF-β1 in patients with prostate cancer treated with radiotherapy are presented in Table 6. A statistically significant positive correlation between patients’ pretreatment IL-6 serum concentrations and maximum fatigue scores was found (*p* = 0.032). A significant positive association was also observed between pretreatment TGF-β1 serum concentrations and maximum acute GU toxicity grades (*p* = 0.036).

**Table 6:** Correlations between maximum acute genitourinary (GU) toxicity grade, maximum acute gastrointestinal (GI) toxicity grade, maximum fatigue score, and serum concentrations of IL-6 and TGF-β1 in patients with prostate cancer treated with radiotherapy

Variable 1	Variable 2	Correlation coefficient	*p*
Maximum GU toxicity grade	Maximum GI toxicity grade	0.09	0.594
Maximum GU toxicity grade	Maximum fatigue score	0.15	0.353
Maximum GI toxicity grade	Maximum fatigue score	0.26	0.117
Pretreatment IL-6 concentration	Maximum GU toxicity grade	0.31	0.054
Pretreatment IL-6 concentration	Maximum GI toxicity grade	0.10	0.548
Pretreatment IL-6 concentration	Maximum fatigue score	0.35	**0.032**
IL-6 concentration after the 25^th^ fraction	Maximum GU toxicity grade	0.21	0.199
IL-6 concentration after the 25^th^ fraction	Maximum GI toxicity grade	0.08	0.640
IL-6 concentration after the 25^th^ fraction	Maximum fatigue score	0.29	0.069
Pretreatment TGF-β1 concentration	Maximum GU toxicity grade	0.34	**0.036**
Pretreatment TGF-β1 concentration	Maximum GI toxicity grade	0.06	0.724
Pretreatment TGF-β1 concentration	Maximum fatigue score	0.19	0.257
TGF-β1 concentration after the 25^th^ fraction	Maximum GU toxicity grade	0.31	0.052
TGF-β1 concentration after the 25^th^ fraction	Maximum GI toxicity grade	0.10	0.535
TGF-β1 concentration after the 25^th^ fraction	Maximum fatigue score	0.29	0.074

**Fig. 5:**
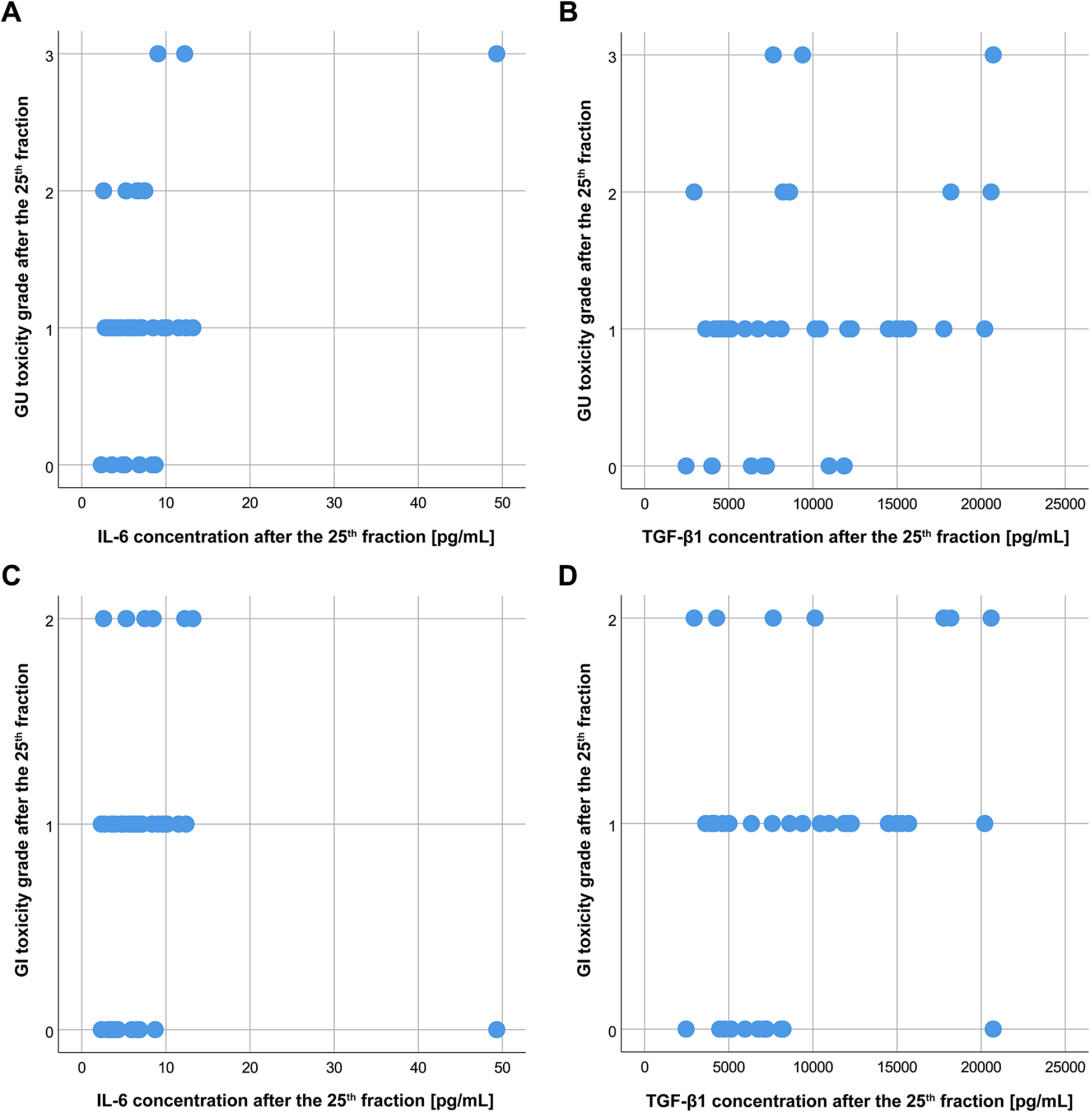
Correlations between acute genitourinary (GU) toxicity grade (**A**, **B**), acute gastrointestinal (GI) toxicity grade (**C**, **D**), and serum concentrations of IL-6 and TGF-β1 after the 25^th^ fraction in patients with prostate cancer treated with radiotherapy

Examination of possible correlations between acute GU toxicity grade, acute GI toxicity grade, fatigue score, and serum concentrations of IL-6 and TGF-β1 after the 25^th^ fraction in patients with prostate cancer treated with radiotherapy, showed a significant positive association between GU toxicity grades after the 25^th^ fraction and fatigue scores after the 25^th^ fraction (*p* = 0.009), as presented in Table 7. A significant positive correlation was observed between IL-6 serum concentrations after the 25^th^ radiotherapy fraction and fatigue scores after the 25^th^ radiotherapy fraction (*p* = 0.042). Furthermore, a statistically significant positive association was demonstrated between TGF-β1 serum concentrations after the 25^th^ radiotherapy fraction and GU toxicity grades after the 25^th^ fraction (*p* = 0.044). A positive correlation between IL-6 serum concentrations after the 25^th^ radiotherapy fraction and GU toxicity grades after the 25^th^ fraction was also found, although it was not statistically significant (*p* = 0.063). The graphs showing correlations between acute GU or GI toxicity grades after the 25^th^ fraction and IL-6 or TGF-β1 concentrations after the 25^th^ fraction are presented in Fig. 5.

**Table 7:** Correlations between acute genitourinary (GU) toxicity grade, acute gastrointestinal (GI) toxicity grade, fatigue score, and serum concentrations of IL-6 and TGF-β1 after the 25^th^ fraction in patients with prostate cancer treated with radiotherapy

Variable 1	Variable 2	Correlation coefficient	*p*
GU toxicity grade after the 25^th^ fraction	GI toxicity grade after the 25^th^ fraction	0.27	0.095
GI toxicity grade after the 25^th^ fraction	Fatigue score after the 25^th^ fraction	0.32	0.051
GU toxicity grade after the 25^th^ fraction	Fatigue score after the 25^th^ fraction	0.41	**0.009**
IL-6 concentration after the 25^th^ fraction	GU toxicity grade after the 25^th^ fraction	0.30	0.063
IL-6 concentration after the 25^th^ fraction	GI toxicity grade after the 25^th^ fraction	0.24	0.147
IL-6 concentration after the 25^th^ fraction	Fatigue score after the 25^th^ fraction	0.33	**0.042**
TGF-β1 concentration after the 25^th^ fraction	GU toxicity grade after the 25^th^ fraction	0.32	**0.044**
TGF-β1 concentration after the 25^th^ fraction	GI toxicity grade after the 25^th^ fraction	0.24	0.145
TGF-β1 concentration after the 25^th^ fraction	Fatigue score after the 25^th^ fraction	0.24	0.149

Investigation of possible relations between changes in circulating IL-6 or TGF-β1 levels and acute GU and GI toxicity grades across radiotherapy performed by univariate analyses controlled for type of radiotherapy, showed no significant associations (Supplementary Table 1). The univariate analysis for late radiotoxicity data demonstrated an absence of associations between changes in IL-6 or TGF-β1 serum levels and late GU and GI toxicity grades assessed between the 3^rd^ and 30^th^ month after radiotherapy.

The levels of IL-6 and TGF-β1 determined before radiotherapy and after the 25^th^ fraction were examined for possible correlations with a maximum grades of late GU and GI toxicity. These results are presented in Supplementary Table 2. No significant correlations of IL-6 or TGF-β1 levels with late GU or GI radiotoxicity were found. However, a negative correlation very close to being significant between TGF-β1 concentrations after the 25^th^ radiotherapy fraction and maximum grades of late GI toxicity was observed (*p* = 0.052). In addition, changes in IL-6 concentrations or TGF-β1 concentrations were not associated with maximum grades of late GU or GI toxicity, as confirmed by univariate analyses controlled for type of radiotherapy.

## Discussion

Identification and integration of multiple treatment-related, clinical, and biological factors affecting the severity of radiotoxicity and fatigue intensity in patients with prostate cancer treated with radiotherapy might be useful for stratification of patients into subgroups with higher and lower risk of developing adverse effects, individual optimization of radiotherapy, and long-term enhancement of the patient’s quality of life. The results of our study showed the statistically significant positive association between grades of acute GU toxicity across radiotherapy and the presence of diabetes in patients, which is in line with observations reported by other studies which identified diabetes as an important factor affecting normal tissue reactions to radiotherapy for prostate cancer [12, 37–40]. The study by Stankovic et al. showed that diabetes was a significant predictive factor of acute GU radiotoxicity grade [12]. The presence of diabetes in prostate cancer patients had a significant effect on the occurrence of acute and late GU toxicities [12]. The effects of diabetes on the occurrence of radiotoxicity in cancer patients could be explained by long-term pathological changes, such as impaired immune system, endothelial dysfunction causing tissue ischemia, impaired wound healing, and tissue repair after radiotherapy [37, 41, 42].

Chronic hypertension, smoking status, alcohol consumption, and previous abdominal operation, were not significantly associated with changes in acute GU toxicity grades in our patient cohort. In contrast to these results, Stankovic et al. reported that smoking, previous abdominal surgery, and the use of diuretics had significant effects on the occurrence of the higher grade of acute GU toxicity [12]. The observed discrepancies in results between the two studies could be attributed to differences in the patient’s, clinical, tumor, and radiation treatment characteristics, and the size of the patient groups. The pretreatment GU problems, previous resection of the prostate or of a bladder tumor, and the presence of acute GU toxicity have been suggested as risk factors for GU morbidity in patients with prostate cancer [43 and references cited therein].

None of the examined patient’s individual and clinical factors were associated with changes in acute GI toxicity grades in our cohort. In contrast to our results, Alashkham et al. showed that prostate cancer patients with diabetes had a significantly higher grades of acute rectal radiotoxicity (proctitis) [38]. Another study demonstrated that patients with diabetes did not have a higher risk of late grade 2 or 3 GI toxicity after radiotherapy for prostate cancer [37]. Similar to the results of the present research, alcohol consumption was not identified as a significant factor involved in the risk for development of high-grade acute GI toxicity after radiotherapy for prostate cancer [31]. The advanced age, larger rectal volume, previous abdominal surgery, the concomitant use of androgen deprivation, preexisting diabetes mellitus, hemorrhoids, or inflammatory bowel disease, have been identified as the most important risk factors for acute and late GI toxicities after radiotherapy for prostate cancer ( [43] and references cited therein]).

Among five examined individual and clinical parameters, smoking was the only parameter that significantly affected the maximum fatigue scores throughout the course of radiotherapy. The effect of cigarette smoking on fatigue may be attributed at least in part to oxidative stress and low-grade systemic inflammation caused by toxic compounds present in smoke and having a negative influence on lung, muscle, and cardiovascular functions [44]. Smoking had been linked with urgency, as a common urinary tract symptom in older people, which may cause tiredness [45].

The grades of radiation-induced GU and GI toxicity after the 25^th^ radiotherapy fraction were positively associated with fatigue scores after the 25^th^ fraction. Our observation may suggest that radiotherapy-caused acute inflammation and injury of the lower GI and GU tract causing complications may contribute to fatigue symptoms. Our finding is in agreement with the study which reported greater urinary urgency associated with greater fatigue increase in patients with prostate cancer receiving radiotherapy [46].

Differential profiles of cytokine expression assessed before and throughout the course of radiotherapy have been suggested as a possible promising approach for the prediction of each patient’s normal tissue radiosensitivity and personalized radiotherapy plan. The IL-6 serum levels were significantly increased in patients with prostate cancer after the 25^th^ radiotherapy fraction in comparison with those levels determined before radiotherapy. Results from seven research studies showed altered cytokine levels in postradiotherapy blood samples, compared with those levels in preradiotherapy blood samples of prostate cancer patients [26, 47–52] and these findings are in accordance with our results. For example, Johnke et al. reported that TGF-β, IL-1β, and IL-6 levels were significantly increased during radiotherapy compared to the cytokine concentrations in blood before radiotherapy [47]. To investigate the possible role of circulating levels of cytokines IL-6 and TGF-β1 in the occurrence and severity of adverse effects of radiotherapy for prostate cancer, we examined the possible relationships between serum concentrations of IL-6 and TGF-β1 determined before radiotherapy and after the 25^th^ radiotherapy fraction, and acute GU and GI radiotoxicity, as well as fatigue. The significant positive correlation was observed between pretreatment TGF-β1 serum levels and maximum GU toxicity grades during the course of radiotherapy. TGF-β1 serum levels after the 25^th^ fraction were positively correlated with maximum GU toxicity grades, although this correlation was not statistically significant, but showed a tendency towards significance. Also, the statistically significant positive association was demonstrated between TGF-β1 serum concentrations after the 25^th^ radiotherapy fraction and GU toxicity grades after the 25^th^ fraction. The IL-6 serum levels before radiotherapy were positively correlated with maximum GU toxicity grades in our patient cohort. The positive association between IL-6 serum levels after the 25^th^ radiotherapy fraction and acute GU radiotoxicity grades at the same time point was found. The determined associations were not statistically significant, but they were very close reaching significance. These results confirm the possible clinical value of determining levels of cytokines for the prediction of the risk for development of radiotherapy-induced normal tissue toxicity, as suggested by the growing body of literature data [26, 27, 53, 54]. The relationship between serum concentrations of IL-6 and acute GU radiotoxicity observed in the present research is consistent with our previous investigation [27]. Promising results for IL-6 as a potential predictor of adverse reactions to radiotherapy derived from our two studies are opposite to the study which reported that changes in IL-6 circulating levels over baseline were not connected with increased GU radiotoxicity [26].

To the best of our knowledge, the present study is the first to report the serum levels of TGF-β1 determined before radiotherapy and after the 25^th^ radiotherapy fraction in prostate cancer patients to be significantly positively associated with acute GU radiotoxicity symptoms. In contrast to our results, the study by Singh et al. showed that prostate cancer patients treated with intensity-modulated radiotherapy who had a higher grade of acute GU radiotoxicity had lower plasma TGF-β1 concentrations at the end of therapy and 3 months after therapy [55]. The same inverse proportion was observed for concentrations of TGF-β1 and grades of acute GI radiotoxicity in this patient cohort [55]. The increased levels of circulating TGF-β1 had been associated with the risk of pulmonary radiotoxicity in patients with lung cancer [56]. In addition, the increased pretreatment plasma levels of TGF-β1 were found to be positively correlated with the development of radiation-induced fibrosis in breast cancer patients [57, 58]. The results of our research might suggest the possible role of TGF-β1 in biological pathways underlying the development of acute GU radiotoxicity in prostate cancer patients, which should be explored further.

Serum levels of IL-6 and TGF-β1 were not statistically significantly associated with maximum grades of late GU toxicity or maximum grades of GI toxicity in our patient group. However, the negative association close to being significant was observed between TGF-β1 levels after the 25^th^ fraction and maximum late GI toxicity grades. These results could be due to the sample size and relatively short time of up to 30 months for assessing late radiotoxicity. Determination of the post-treatment circulating levels of IL-6 and TGF-β1 in prostate cancer patients after 1,3,6, and 12 months after the end of radiotherapy could be important for evaluating their potential significance for predicting late GU and GI radiotoxicity and will be the focus of further studies with a larger number of patients.

Considering the possible influence of cytokines on fatigue, the pretreatment serum levels of IL-6 were significantly positively associated with maximum fatigue scores. It is important to note that serum levels of IL-6 and TGF-β1 measured after the 25^th^ radiotherapy fraction were positively correlated with maximum fatigue scores, although these results were not statistically significant. The possible influence of IL-6 on symptoms of fatigue was further confirmed by significant positive correlation between serum levels of IL-6 and fatigue scores determined after the 25^th^ fraction. Our results confirm the relationship between circulating levels of IL-6 and fatigue intensity in prostate cancer patients undergoing curative radiotherapy and may contribute to further understanding of biological factors underlying fatigue. The increase in IL-6 and fatigue across radiotherapy was already demonstrated in prostate cancer patients, although the IL-6 increase was not significantly associated with fatigue levels [51]. The relatively small number of patients in the pilot study reported by Holliday and colleagues may contribute to the inability of this study to detect significant associations between longitudinal cytokine levels and fatigue scores. Another study also reported the absence of correlations between pretreatment and post-treatment serum concentrations of IL-4, IL-6, IL-10, and TNF-α, and fatigue symptoms in a group of 29 prostate cancer patients treated with radiotherapy [50]. Bower and colleagues did not find associations between serum levels of proinflammatory cytokines IL-1β and IL-6, and fatigue severity in patients with breast cancer and patients with prostate cancer treated with radiotherapy [24]. In addition, IL-6 serum levels measured during radiotherapy in prostate cancer patients were positively correlated with enhancement of fatigue symptoms, but this result was not statistically significant (*p* = 0.089) [52]. This relationship was not found for pretreatment IL-6 levels [52].

The potential role of TGF-β1, as a substantial component of cytokine signature, in the occurrence of fatigue, is suggested by a study of Montoya et al. which demonstrated increased serum concentrations of TGF-β1 in patients with chronic fatigue syndrome when compared with those concentrations in healthy controls [59]. Another study found a negative correlation between TGF-β and fatigue intensity in patients with pancreatic cancer [60]. A significant moderate correlation between TGF-β1 serum levels and cognitive function was observed in patients with advanced cancer [61]. The possible relationship between circulating levels of TGF-β1 and fatigue revealed in our study needs to be examined in future studies on a larger cohort of prostate cancer patients receiving radiotherapy.

A recent study reported an establishment of a predictive model generated by machine learning that included pretreatment cytokine levels (IL-8 and CCL2), clinical factor (hypertension), and radiation dosimetric factor (mean lung dose) for radiation pneumonitis grade higher or equal to 2 in patients with non-small lung cancer receiving radiotherapy [62]. The study highlights the need for integration of circulating cytokine signatures and multiple clinical factors in machine learning models for the identification of cancer patients at higher risk for developing adverse normal tissue reactions to radiotherapy, as suggested by our research studies on cytokine profiles in patients with prostate cancer treated with radiotherapy.

## Conclusions

The present research showed significant positive correlations between pretreatment concentrations of circulating TGF-β1 and maximum GU toxicity grades and between TGF-β1 concentrations measured after the 25^th^ fraction and GU toxicity grades after this fraction, in patients with prostate cancer undergoing curative radiotherapy. The presence of diabetes in patients was significantly associated with higher acute GU toxicity grades across radiotherapy. Regarding the influence of cytokines on radiotherapy-induced fatigue, the pretreatment IL-6 concentrations were significantly positively associated with maximum fatigue scores during radiotherapy in addition to the significant positive association between IL-6 concentrations and fatigue scores after the 25^th^ fraction. Smoking was identified as a factor that significantly affected the intensity of fatigue symptoms in our patient cohort. The levels of circulating IL-6 were significantly higher at the 25^th^ radiotherapy fraction when compared with those levels before radiotherapy. Taken together, the results of our study demonstrate that circulating levels of IL-6 and TGF-β1 might influence the severity of acute GU radiotoxicity and fatigue in patients with prostate cancer treated with radiotherapy. Combining clinical parameters, patient’s individual characteristics, and circulating cytokine levels, especially IL-6 and TGF-β1, in prediction models might be useful for the prediction of adverse normal tissue reactions to radiotherapy in patients with prostate cancer. To develop and validate these predictive models, further studies with larger patient cohorts are required.

## Supplementary Information

**Additional file 1.**

## Data Availability

All data generated or analyzed during this study are included in this published article. Raw and processed data are stored in the laboratories and are available from the corresponding author on reasonable request.

## Abbreviations

GU: Genitourinary
GI: Gastrointestinal
CRF: Cancer-related fatigue
NCCN: National Comprehensive Cancer Network
3DCRT: 3D conformal radiotherapy
VMAT: Volumetric Modulated Arc Therapy
EPIC: Expanded Prostate Cancer Index Composite
BFI: Brief Fatigue Inventory
SV: Seminal vesicles
P: Prostate-only group
WPRT: Whole pelvic radiotherapy
CTV: Clinical target volume
PTV: Planning target volume
ICRU: International Commission on Radiation Units and Measurements
LNN: Lymph nodes

## References

[1] Sung H, Ferlay J, Siegel RL, Laversanne M, Soerjomataram I, Jemal A (2021). Global Cancer Statistics 2020: GLOBOCAN Estimates of Incidence and Mortality Worldwide for 36 Cancers in 185 Countries. CA Cancer J Clin.

[2] American Cancer Society (2011). Cancer Facts and Figures 2011.

[3] Johansson E, Steineck G, Holmberg L, Johansson JE, Nyberg T, Ruutu M (2011). Long-term quality-of-life outcomes after radical prostatectomy or watchful waiting: the Scandinavian Prostate Cancer Group-4 randomised trial. Lancet Oncol.

[4] Luo HC, Cheng HH, Lin GS, Fu ZC, Li DS (2013). Intensity-modulated radiotherapy combined with endocrine therapy for intermediate and advanced prostate cancer: long-term outcome of Chinese patients. Asian Pac J Cancer Prev.

[5] Penson DF (2007). Quality of life after therapy for localized prostate cancer. Cancer J.

[6] Foroudi F, Tyldesley S, Barbera L, Huang J, Mackillop WJ (2003). Evidence-based estimate of appropriate radiotherapy utilization rate for prostate cancer. Int J Radiat Oncol Biol Phys.

[7] Burnet NG, Johansen J, Turesson I, Nyman J, Peacock JH. Describing patients’ normal tissue reactions: Concerning the possibility of individualising radiotherapy dose prescriptions based on potential predictive assays of normal tissue radiosensitivity. Steering Committee of the BioMed2European Union Concerted Action Programme on the development of predictive tests of normal tissue response to radiation therapy. Int J Cancer. 1998;79:606–13. 10.1002/(sici)1097-0215(19981218)79:6<606::aid-ijc9>3.0.co;2-y 9842969

[8] Wang K, Tepper JE (2021). Radiation therapy-associated toxicity: Etiology, management, and prevention. CA Cancer J Clin.

[9] West CM, Barnett GC (2011). Genetics and genomics of radiotherapy toxicity: towards prediction. Genome Med.

[10] Begg AC (2006). Can the severity of normal tissue damage after radiation therapy be predicted?. PLoS Med.

[11] Wang D, Zhang Q, Eisenberg BL, Kane JM, Li XA, Lucas D (2015). Significant reduction of late toxicities in patients with extremity sarcoma treated with image-guided radiation therapy to a reduced target volume: Results of radiation therapy oncology group RTOG-0630 Trial. J Clin Oncol.

[12] Stankovic V, Džamic Z, Pekmezovic T, Tepavcevic DK, Dozic M, Saric M (2016). Acute and Late Genitourinary Toxicity after 72 Gy of Conventionally Fractionated Conformal Radiotherapy for Localised Prostate Cancer: Impact of Individual and Clinical Parameters. Clin Oncol (R Coll Radiol).

[13] Valdagni R, Vavassori V, Rancati T, Fellin G, Baccolini M, Bianchi C (2012). Increasing the risk of late rectal bleeding after high-dose radiotherapy for prostate cancer: The case of previous abdominal surgery. Results from a prospective trial. Radiother Oncol..

[14] Fellin G, Rancati T, Fiorino C, Vavassori V, Antognoni P, Baccolini M (2014). Long term rectal function after high-dose prostate cancer radiotherapy: Results from a prospective cohort study. Radiother Oncol.

[15] Fiorino C, Rancati T, Fellin G, Vavassori V, Cagna E, Casanova Borca V (2012). Late fecal incontinence after high-dose radiotherapy for prostate cancer: Better prediction using longitudinal definitions. Int J Radiat Oncol Biol Phys.

[16] Hunter GK, Reddy CA, Klein EA, Kupelian P, Angermeier K, Ulchaker J, et al. Long-term (10-Year) gastrointestinal and genitourinary toxicity after treatment with external beam radiotherapy, radical prostatectomy, or brachytherapy for prostate cancer. Prostate Cancer. 2012;2012:853487. 10.1155/2012/853487 PMC3345236 22577562

[17] Cella L, D’Avino V, Liuzzi R, Conson M, Doria F, Faiella A, et al. Multivariate normal tissue complication probability modeling of gastrointestinal toxicity after external beam radiotherapy for localized prostate cancer. Radiat Oncol. 2013;8:221. 10.1186/1748-717X-8-221 PMC3852304 24053357

[18] Langston B, Armes J, Levy A, Tidey E, Ream E (2013). The prevalence and severity of fatigue in men with prostate cancer: a systematic review of the literature. Support Care Cancer.

[19] Storey DJ, McLaren DB, Atkinson MA, Butcher I, Frew LC, Smyth JF, Sharpe M (2012). Clinically relevant fatigue in men with hormone-sensitive prostate cancer on long-term androgen deprivation therapy. Ann Oncol.

[20] Wang XS, Zhao F, Fisch MJ, O’Mara AM, Cella D, Mendoza TR, Cleeland CS. Prevalence and characteristics of moderate to severe fatigue: a multicenter study in cancer patients and survivors. Cancer. 2014;120(3):425–32. 10.1002/cncr.28434 PMC3949157 24436136

[21] Saligan LN, Olson K, Filler K, Larkin D, Cramp F, Yennurajalingam S (2015). The biology of cancer-related fatigue: a review of the literature. Support Care Cancer.

[22] Provatopoulou X, Athanasiou E, Gounaris A (2008). Predictive markers of radiation pneumonitis. Anticancer Res.

[23] Ding NH, Li JJ, Sun LQ (2013). Molecular mechanisms and treatment of radiation-induced lung fibrosis. Curr Drug Targets.

[24] Bower JE, Ganz PA, Tao ML, Hu W, Belin TR, Sepah S (2009). Inflammatory biomarkers and fatigue during radiation therapy for breast and prostate cancer. Clin Cancer Res.

[25] Bower JE, Lamkin DM (2013). Inflammation and cancer-related fatigue: mechanisms, contributing factors, and treatment implications. Brain Behav Immun..

[26] Christensen E, Pintilie M, Evans KR, Lenarduzzi M, Ménard C, Catton CN (2009). Longitudinal cytokine expression during IMRT for prostate cancer and acute treatment toxicity. Clin Cancer Res.

[27] Stanojković TP, Matić IZ, Petrović N, Stanković V, Kopčalić K, Besu I (2020). Evaluation of cytokine expression and circulating immune cell subsets as potential parameters of acute radiation toxicity in prostate cancer patients. Sci Rep.

[28] Peeters ST, Heemsbergen WD, van Putten WL, Slot A, Tabak H, Mens JW (2005). Acute and late complications after radiotherapy for prostate cancer: results of a multicenter randomized trial comparing 68 Gy to 78 Gy. Int J Radiat Oncol Biol Phys.

[29] Wei JT, Dunn RL, Litwin MS, Sandler HM, Sanda MG (2000). Development and validation of the expanded prostate cancer index composite (EPIC) for comprehensive assessment of health-related quality of life in men with prostate cancer. Urology.

[30] Mendoza TR, Wang XS, Cleeland CS, Morrissey M, Johnson BA, Wendt JK, Huber SL (1999). The rapid assessment of fatigue severity in cancer patients: use of the Brief Fatigue Inventory. Cancer.

[31] Stankovic V, Nikitovic M, Pekmezovic T, Pekmezovic D, Kisic Tepavcevic D, Stefanovic Djuric A, Saric M (2016). Toxicity of the lower gastrointestinal tract and its predictive factors after 72 Gy conventionally fractionated 3D conformal radiotherapy of localized prostate cancer. J BUON.

[32] Roach M (1993). Equations for predicting the pathologic stage of men with localized prostate cancer using the preoperative prostate specific antigen (PSA) and Gleason score. J Urol.

[33] ICRU Report 50 (1993). Prescribing, recording, and reporting photon beam therapy.

[34] ICRU Report 62 (1999). Prescribing, recording and reporting photon beam therapy (supplement to ICRU report 50).

[35] International Commission on Radiation Units and Measurements. Prescribing, Recording, and Reporting Photon-Beam Intensity Modulated Radiation Therapy (IMRT). ICRU Report 83. Vol. 10, Journal of the ICRU. Oxford University Press; 2010.

[36] Marks LB, Yorke ED, Jackson A, Ten Haken RK, Constine LS, Eisbruch A (2010). Use of normal tissue complication probability models in the clinic. Int J Radiat Oncol Biol Phys.

[37] Kalakota K, Liauw SL (2013). Toxicity after external beam radiotherapy for prostate cancer: an analysis of late morbidity in men with diabetes mellitus. Urology.

[38] Alashkham A, Paterson C, Hubbard S, Nabi G (2017). What is the impact of diabetes mellitus on radiation induced acute proctitis after radical radiotherapy for adenocarcinoma prostate? A prospective longitudinal study. Clin Transl Radiat Oncol.

[39] Dağdelen M, Barlas C, Yıldırım C, Çavdar Karaçam S, Öner Dinçbaş HF (2021). Effect of Diabetes Mellitus and Metformin Usage on Treatment Outcomes and Side Effects on Prostate Cancer Treated with Radical Radiotherapy. Bull Urooncol.

[40] Yahya N, Ebert MA, Bulsara M, Haworth A, Kennedy A, Joseph DJ, Denham JW (2015). Dosimetry, clinical factors and medication intake influencing urinary symptoms after prostate radiotherapy: An analysis of data from the RADAR prostate radiotherapy trial. Radiother Oncol.

[41] Turina M, Fry DE, Polk HC (2005). Acute hyperglycemia and the innate immune system: clinical, cellular, and molecular aspects. Crit Care Med.

[42] Stone HB, Coleman CN, Anscher MS, McBride WH (2003). Effects of radiation on normal tissue: consequences and mechanisms. Lancet Oncol.

[43] Budäus L, Bolla M, Bossi A, Cozzarini C, Crook J, Widmark A, Wiegel T (2012). Functional outcomes and complications following radiation therapy for prostate cancer: a critical analysis of the literature. Eur Urol.

[44] Darabseh MZ, Maden-Wilkinson TM, Welbourne G, Wüst RCI, Ahmed N, Aushah H (2021). Fourteen days of smoking cessation improves muscle fatigue resistance and reverses markers of systemic inflammation. Sci Rep.

[45] Nuotio M, Jylhä M, Koivisto AM, Tammela TL (2001). Association of smoking with urgency in older people. Eur Urol.

[46] Chao HH, Doucette A, Raizen DM, Vapiwala N (2018). Factors associated with fatigue in prostate cancer (PC) patients undergoing external beam radiation therapy (EBRT). Pract Radiat Oncol.

[47] Johnke RM, Edwards JM, Evans MJ, Nangami GN, Bakken NT, Kilburn JM (2009). Circulating cytokine levels in prostate cancer patients undergoing radiation therapy: influence of neoadjuvant total androgen suppression. In Vivo.

[48] Kovacs CJ, Daly BM, Evans MJ, Johnke RM, Lee TK, Karlsson UL (2003). Cytokine profiles in patients receiving wide-field + prostate boost radiotherapy (xRT) for adenocarcinoma of the prostate. Cytokine.

[49] Tanji N, Kikugawa T, Ochi T, Taguchi S, Sato H, Sato T (2015). Circulating Cytokine Levels in Patients with Prostate Cancer: Effects of Neoadjuvant Hormonal Therapy and External-beam Radiotherapy. Anticancer Res.

[50] Dirksen SR, Kirschner KF, Belyea MJ (2014). Association of symptoms and cytokines in prostate cancer patients receiving radiation treatment. Biol Res Nurs.

[51] Holliday EB, Dieckmann NF, McDonald TL, Hung AY, Thomas CR, Wood LJ (2016). Relationship between fatigue, sleep quality and inflammatory cytokines during external beam radiation therapy for prostate cancer: A prospective study. Radiother Oncol.

[52] Feng LR, Wolff BS, Lukkahatai N, Espina A, Saligan LN (2017). Exploratory Investigation of Early Biomarkers for Chronic Fatigue in Prostate Cancer Patients Following Radiation Therapy. Cancer Nurs.

[53] Singh J, Sohal SS, Lim A, Duncan H, Thachil T, De Ieso P (2019). Cytokines expression levels from tissue, plasma or serum as promising clinical biomarkers in adenocarcinoma of the prostate: a systematic review of recent findings. Ann Transl Med.

[54] Sprung CN, Forrester HB, Siva S, Martin OA (2015). Immunological markers that predict radiation toxicity. Cancer Lett.

[55] Singh J, Sohal SS, Ahuja K, Lim A, Duncan H, Thachil T, De Ieso P (2020). Investigation of circulatory cytokines in patients undergoing intensity-modulated radiotherapy (IMRT) for adenocarcinoma of the prostate and association with acute RT-induced toxicity: a prospective clinical study. Cytokine.

[56] Zhao L, Wang L, Ji W, Wang X, Zhu X, Hayman JA (2009). Elevation of plasma TGF-beta1 during radiation therapy predicts radiation-induced lung toxicity in patients with non-small-cell lung cancer: a combined analysis from Beijing and Michigan. Int J Radiat Oncol Biol Phys.

[57] Li C, Wilson PB, Levine E, Barber J, Stewart AL, Kumar S (1999). TGF-beta1 levels in pre-treatment plasma identify breast cancer patients at risk of developing post-radiotherapy fibrosis. Int J Cancer.

[58] Boothe DL, Coplowitz S, Greenwood E, Barney CL, Christos PJ, Parashar B (2013). Transforming growth factor β-1 (TGF-β1) is a serum biomarker of radiation induced fibrosis in patients treated with intracavitary accelerated partial breast irradiation: preliminary results of a prospective study. Int J Radiat Oncol Biol Phys.

[59] Montoya JG, Holmes TH, Anderson JN, Maecker HT, Rosenberg-Hasson Y, Valencia IJ (2017). Cytokine signature associated with disease severity in chronic fatigue syndrome patients. Proc Natl Acad Sci U S A.

[60] Breitbart W, Rosenfeld B, Tobias K, Pessin H, Ku GY, Yuan J, Wolchok J (2014). Depression, cytokines, and pancreatic cancer. Psychooncology.

[61] Paulsen Ø, Laird B, Aass N, Lea T, Fayers P, Kaasa S, Klepstad P (2017). The relationship between pro-inflammatory cytokines and pain, appetite and fatigue in patients with advanced cancer. PLoS ONE.

[62] Yu H, Wu H, Wang W, Jolly S, Jin JY, Hu C, Kong FS (2019). Machine Learning to Build and Validate a Model for Radiation Pneumonitis Prediction in Patients with Non-Small Cell Lung Cancer. Clin Cancer Res.

