# Supplementary material for: Circulating levels of IL-6 and TGF-β1 in patients with prostate cancer undergoing radiotherapy: associations with acute radiotoxicity and fatigue symptoms
Source: BMC Cancer. 2022 Nov 11;22:1167. doi: 10.1186/s12885-022-10255-6 (PMC9652872; doi:10.1186/s12885-022-10255-6)
Supplement: Supplementary file 1 — Additional file 1. [file 12885_2022_10255_MOESM1_ESM.pdf]

Supplementary files

Supplementary Table 1: Associations between changes of IL-6 and TGF-β1 and genitourinary (GU) and gastrointestinal (GI) toxicity grades over time

**Parameters**	**Univariate analysis***	
	**b**	***p***
Changes of IL-6 and acute GU toxicity grades	0.017	0.559
Changes of TGF-β1 and acute GU toxicity grades	<-0.001	0.672
Changes of IL-6 and acute GI toxicity grades	0.013	0.558
Changes of TGF-β1 and acute GI toxicity grades	<0.001	0.305
Changes of IL-6 and late GU toxicity grades	0.009	0.750
Changes of TGF-β1 and late GU toxicity grades	<0.001	0.351
Changes of IL-6 and late GI toxicity grades	0.018	0.105
Changes of TGF-β1 and late GI toxicity grades	<-0.001	0.656

* multilevel ordinal regression models with the degree of toxicity as the dependent variable, controlled for type of radiotherapy

Supplementary Table 2: Correlations between maximum late genitourinary (GU) toxicity grade, maximum late gastrointestinal (GI) toxicity grade, and serum concentrations of IL-6 and TGF-β1 in patients with prostate cancer treated with radiotherapy

**Variable 1**	**Variable 2**	**Correlation coefficient**	***p***
Pretreatment IL-6 concentration	Maximum late GU toxicity grade	0.08	0.642
Pretreatment IL-6 concentration	Maximum late GI toxicity grade	0.23	0.156
IL-6 concentration after the 25th fraction	Maximum late GU toxicity grade	0.03	0.872
IL-6 concentration after the 25th fraction	Maximum late GI toxicity grade	0.24	0.138
Pretreatment TGF-β1 concentration	Maximum late GU toxicity grade	-0.21	0.194
Pretreatment TGF-β1 concentration	Maximum late GI toxicity grade	-0.15	0.367
TGF-β1 concentration after the 25th fraction	Maximum late GU toxicity grade	-0.17	0.309
TGF-β1 concentration after the 25th fraction	Maximum late GI toxicity grade	-0.31	0.052
Changes of IL-6 concentrations	Maximum late GU toxicity grade	-0.10	0.534
Changes of IL-6 concentrations	Maximum late GI toxicity grade	0.13	0.419
Changes of TGF-β1 concentrations	Maximum late GU toxicity grade	0.13	0.436
Changes of TGF-β1 concentrations	Maximum late GI toxicity grade	-0.06	0.736
